# Factors Modifying the Amount of Neuroanatomical Overlap between Languages in Bilinguals—A Systematic Review of Neurosurgical Language Mapping Studies

**DOI:** 10.3390/brainsci10120983

**Published:** 2020-12-15

**Authors:** Monika M. Połczyńska, Susan Y. Bookheimer

**Affiliations:** Department of Psychiatry and Biobehavioral Sciences, David Geffen School of Medicine at UCLA, University of California, Los Angeles, CA 90095, USA; sbook@mednet.ucla.edu

**Keywords:** age, bilingual, brain surgery, language mapping, electrical stimulation, fMRI, linguistic distance, language similarity, multilingual, proficiency

## Abstract

Neurosurgery on individuals with lesions around language areas becomes even more complicated when the patient is bilingual. It is thus important to understand the principles that predict the likelihood of convergent versus separate neuroanatomical organization of the first (L1) and the second language (L2) in these individuals. We reviewed all English-language publications on neurosurgical language mapping in bilinguals before January 2020 in three databases (e.g., PubMed). Our search yielded 28 studies with 207 participants. The reviewed data suggest several principles of language organization in bilingual neurosurgical patients: (1) separate cortical areas uniquely dedicated to each language in both anterior and posterior language sites are the rule rather than occasional findings, (2) In cases where there was a convergent neuroanatomical representation for L1 and L2, two factors explained the overlap: an early age of L2 acquisition and a small linguistic distance between L1 and L2 and (3) When L1 and L2 diverged neuroanatomically, more L1-specific sites were identified for early age of L2 acquisition, high L2 proficiency and a larger linguistic distance. This work provides initial evidence-based principles predicting the likelihood of converging versus separate neural representations of L1 and L2 in neurosurgical patients.

## 1. Introduction

Because over half of the world’s population communicates in two or more languages [1], understanding the principles underlying bilingual brain organization on a single-subject level is of importance to neurosurgery. For bilingual patients who continue to require to communicate in both their languages, a major goal of neurosurgery is to map and preserve all their languages [2,3]. There are mixed findings on when and where languages are separate versus overlapping in bilinguals [4,5,6,7,8,9,10]. Thus, there is an important clinical need for evidence-based principles predicting the likelihood of convergent versus divergent neural representations of the first (L1) and the second language (L2) in neurosurgical patients.

Based on neuroimaging studies involving group analyses of healthy individuals, we know that the amount of neuroanatomical overlap between L1 and L2 can be modulated by a variety of factors, including the age of L2 acquisition [11,12], proficiency level of L2 (but also L1) [13], the amount of language exposure to L1 and L2 [14], the manner of L2 acquisition (formal/explicit versus informal/implicit) [15], the linguistic distance between L1 and L2 [16] and the modality of acquisition of L1 and L2 (oral versus signed) [17]. However, most of these studies examined the factors in isolation (i.e., only one or less frequently two factors were examined at a time). Moreover, these findings are not applied as easily at the single-patient level. Subject-level prediction is essential in the neurosurgical population, where knowledge about the organization of each language will direct the use of invasive procedures (e.g., implanting of electrodes to map language) and guide neurosurgery [2,3].

We present a comprehensive systematic review of the factors that have been separately argued to modify the neural architecture in healthy bilinguals. In this work, we solely focused on clinical language mapping studies that included data from bilingual neurosurgical patients. Most of the reviewed reports performed an invasive verification of the representation of L1 and L2 through techniques such as direct cortical stimulation. Invasive techniques offer more precise information on language organization in the brain than neuroimaging methods. While our main focus is studies that performed direct cortical stimulation, we also include reports that used other techniques, such as Wada testing and single-subject functional magnetic resonance imaging (fMRI), for validation. Similarly, our target group is bilingual patients with brain tumors who underwent direct cortical stimulation but we verify the results by extending our analyses to a broader population of bilingual patients who received clinical language mapping (e.g., individuals with epilepsy). Our work provides multiple evidence-based principles that predict the conditions under which languages are more likely to have converging neural representations of L1 and L2 in neurosurgical patients.

## 2. Materials and Methods

**Figure 1:**
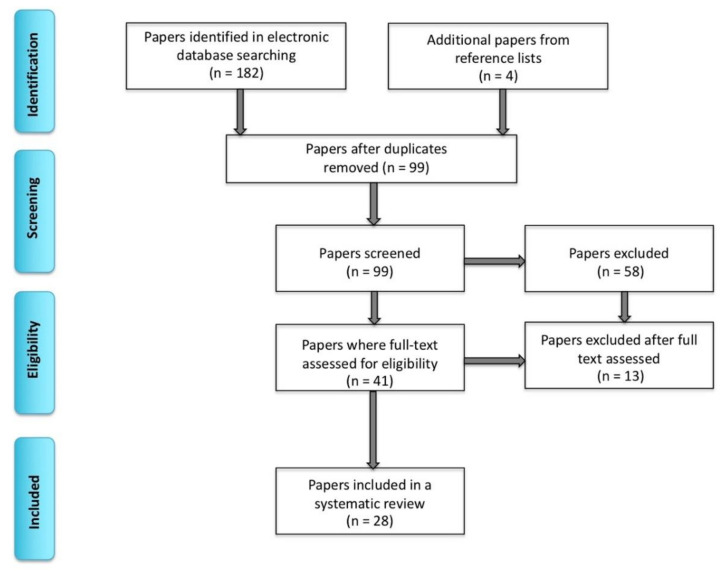
A flow diagram presenting inclusion based of Preferred Items for Systematic Reviews and Meta-Analyses (PRiSMA).

We conducted this systematic review following the Preferred Items for Systematic Reviews and Meta-Analyses [18]. We performed single extraction of literature searches using PubMed, Web of Science and EMBASE databases between November 2018 and January 2020 (including publications ahead of print). The following terms were used: “bilingual,” “multilingual,” “clinical” “language mapping,” “brain tumor,” “epilepsy,” “awake surgery,” “direct electrical stimulation,” “electrocorticography,” (the three terms mentioned last will be collectively referred to as direct electrical stimulation mapping; ESM), “intracarotid amobarbital procedure,” “Wada test,” “fMRI,” “EEG” (electroencephalography), “TMS” (transcranial magnetic stimulation) and “MEG” (magnetoencephalography). After removing duplications, 99 peer-reviewed articles involving human subjects were found through an electronic database search. Among these papers, we only focused on those that included bilingual or multilingual individuals who underwent clinical language mapping for surgical purposes. We did not include papers that only examined healthy volunteers (*n* = 43). Next, we applied the following exclusion criteria included: not bilingual or multilingual (*n* = 12), not pre- or intraoperative language mapping (*n* = 8), a review article (*n* = 1), not in English (*n* = 2), insufficient biomedical, linguistic and methodological information (*n* = 2), not examining languages separately to map areas of overlap (studies on language switching, not examining the amount of separation versus overlap between L1 and L2) (*n* = 1), not an article (*n* = 1) and a positron emission tomography (PET) study (*n* = 1) (see Figure 1 for the inclusion and exclusion criteria). We did not include PET studies because this mapping technique appears to be less sensitive at identifying more subtle differences in the organization of languages in the brain [19]. For detailed biodemographic information about the sample, see Table 1. An example of research results in PubMed is presented in Appendix A. Ultimately, the search totaled 28 papers on clinical language mapping with 207 bilingual patients.

We extracted the following information from the reviewed papers: (1) patient characteristics: lesion etiology, lesion location, age, handedness, gender, presence or absence of aphasia and the age of disease onset, (2) language information: the number of languages beyond L1 (L2+), proficiency level of L2, the age of L2 acquisition, the manner of L2 acquisition, the amount of exposure to L1 and L2, the linguistic distance between L1 and L2 and the modality of acquisition and (3) acquisition techniques of language mapping: the method of acquisition and types of employed languages tasks. We provide a narrative synthesis to summarize and explain both the characteristics and results of the included studies.

As illustrated in Table 1 and Table 2, the reviewed studies used different diagnosis, methods and language tasks. To account for the heterogeneity in the data, we first focused on a common set of studies with a homogenous diagnosis, methods and tasks. Specifically, we only analyzed studies (*n* = 16) (a) on patients with brain tumors, (b) that performed ESM and (c) used an object naming task (with or without other tasks). We then expanded the analyses to other diagnoses (epilepsy, arteriovenous malformation (AVM), aneurysms), methods (fMRI and the Wada test) and tasks to both verify the initial findings and to determine whether there was additional information that was not present in the homogenous study set. Because there was insufficient information and very substantial variability in the sample for our variables of interest, a meta-analysis could not be conducted on the homogenous set of 16 studies. The variables included lesion location, tumor type and grade, patients’ age (pediatric cases were included) and task differences (most of the studies from the set of the 16 reports used an object naming task with a at least one additional task from a divergent pool of 21 language tasks). In addition, there was insufficient information on the possible impact of aphasia on the neuroanatomical representation of L1 and L2 in the reviewed sample. Further, many studies did not include the effect of the linguistic variables of interest (e.g., proficiency level, the manner of L2 acquisition) on the neural organization of L1 and L2 in their results.

Since the majority of patients in the reviewed studies were bilingual, the term “bilingual” will be used throughout the paper except when discussing cases that used more than two languages. Individuals speaking more than two languages (15.9% of the reviewed cases) will be referred to as “multilingual.” Finally, “L2+” will be used as an umbrella term for any language that was acquired after one’s first language, particularly in the context of multilinguals.

## 3. Results

### 3.1. Distinct Versus Overlapping Representations of L1 and L2

The homogenous set of 16 ESM studies focusing on patients with brain tumors all reported at least some areas in the brain with separate representation for L1 and L2. In addition, 14 of the 16 studies found areas where L1 and L2 converged neuroanatomically. The remaining two studies did not identify regions of overlap.

**Figure 2:**
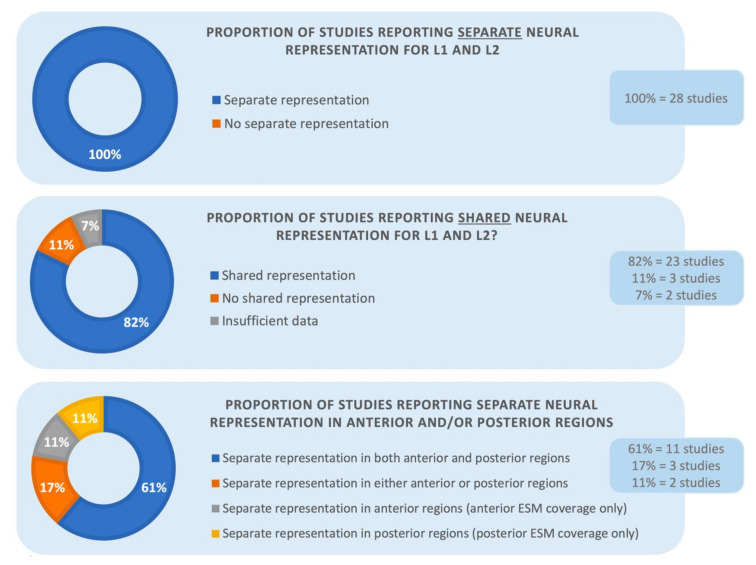
The proportion of studies showing separate versus shared neuroanatomical representation for L1 and L2 in the reviewed studies. The bottom panel additionally presents the proportion of divergent neural organization within the anterior and posterior language sites.

As the next step, we expanded the analyses by adding the remaining 12 studies that included more diverse diagnoses, brain mapping methods and tasks. As presented in Figure 2, of the complete set of 28 studies, all again reported at least some areas in the brain where L1 and L2 were represented separately. In addition, twenty-three studies (82%) identified regions where L1 and L2 had shared neural representation, whereas the other five did not. Of the five studies, three (11%) specifically mentioned that there were no convergent sites and two studies (7%) did not provide information on whether there were any shared language areas in addition to divergent regions. Consistent with this finding, one of the reviewed studies using ESM [2] measured the ratio between divergent and co-localizing language sites in 13 patients: separate language areas constituted the majority (66%) of the mapped regions with ESM, compared to common language sites (34%).

### 3.2. Regional Specificity of Language Overlap

Among the 16 homogenous studies, thirteen examined language-specific regions in the frontal versus posterior language sites. Twelve of the thirteen studies identified divergent representation for each examined language in both anterior and posterior language areas. Four additional studies were restricted to one of the areas due to limited ESM coverage—two reports assessed language function only in the anterior language areas and two in the posterior areas. These studies also noted separate language organization in the respective regions.

The results remained similar for the complete set of 28 studies. Fourteen of the 28 studies focused on language-specific areas in the frontal versus posterior language areas. Eleven of the 14 studies (61%) reported a distinct organization for each examined language in both anterior and posterior language areas. Three studies (17%) found separate representation for L1 and L2 in either the anterior versus posterior language sites. Once again, four additional studies had restricted ESM coverage within language sites and they showed separate neural representation for L1 and L2 in their respective areas (anterior and posterior, 11% each, respectively). The bottom panel of Figure 2 presents the results in percentiles. These findings are clinically significant because they highlight the need to map L1 and L2 both anteriorly and posteriorly for surgical purposes.

### 3.3. Factors Modifying the Amount of Overlap between L1 and L2

We found sufficient data to draw conclusions on three factors: age of L2 acquisition, proficiency level of L2 and the linguistic distance between L1 and L2 (see the sections below). There was insufficient data to determine the effects of the following three factors on the amount of neuroanatomical co-localization between L1 and L2: the amount of exposure to L1 and L2, whether languages are acquired formally or informally or whether the languages are oral or signed.

#### 3.3.1. Age of Acquisition

When it was possible, we defined early acquisition as below age five and late acquisition as above age five based on neuroimaging developmental data [42]. However, some of the reviewed studies applied their own cutoffs (in two studies, the cutoff age was age six and in two studies it was age seven, see Table 3). The studies did not provide sufficient data for us to modify their cutoff to age five. Among 256 instances of L2+ examined in the 28 reviewed studies, 107 were acquired early and 127 were learned late in life. The age of acquisition was not reported in one study (22 instances of L2+) (see Table 3).

The majority of the reviewed studies did not explicitly evaluate the impact of the age of L2 acquisition on the degree of neuroanatomical overlap between L1 and L2 in the brain. However, three ESM studies and one fMRI study have provided compelling evidence that languages are more likely to co-localize in the brain in patients who acquired their L2 earlier in life. Those studies included both bi- and multilingual patients. Specifically, Walker et al. [23] reported that in a patient who had acquired both of their languages at infancy, stimulation of the same sites resulted in anomia in L1 and L2. In the remaining four individuals in whom anomia was identified, L2 had been learned late (between ages five and six). These patients displayed some overlap between L1 and L2 but with a concurrent higher degree of separation between the languages (i.e., anomia in either L1 or L2). In their ESM study, Fernández-Coello et al. [2] observed that considering the number of positive overlapping language regions for L1 and L2 in early versus late highly proficient bi-/multilinguals, the former group of bi-/multilinguals had a greater overlapping representation compared to the latter group. In a similar vein, Połczyńska et al. [33] demonstrated that, in a multilingual patient, earlier acquired languages (L1 and L2) were more affected by aphasia subsequent to a high-grade tumor than later acquired languages (L3 and L4), despite a high level of proficiency in all the L2+. Thus, the earlier acquired languages appeared to have a more overlapping representation, whereas the later learned languages diverged neuroanatomically. In sum, the three reports demonstrated that L1 and L2+ are more likely to converge in early bi- and multilinguals and diverge and late bi- and multilinguals.

While the three studies focused on within-hemisphere localization of L1 and L2, one fMRI report [20] examined hemispheric lateralization of L1 and L2. The study compared early simultaneous bilinguals with monolinguals (“simultaneous bilinguals” are individuals who acquired their L1 and L2 at the same time). All the participants were neurosurgical patients who received functional language fMRI before their brain surgery. The laterality index values based on the amount of functional activation in each hemisphere did not significantly differ for L1 and L2 in the bilingual group. Interestingly, the bilingual patients were found to have significantly lower laterality index values for their L1, compared with the monolingual patients. Note that the volume of activation for L1 in the left hemisphere was similar in both groups, while the volume of activation in the right hemisphere was more robust in the bilingual patients, indicating that the reduced laterality indices were explained by right hemisphere recruitment during language tasks.

The remaining paragraphs of this section will report results from bi- and multilingual patients. Since in several studies bilinguals and multilinguals were clustered as a single group, we will use the term “L2” to describe L2 in bilinguals and languages beyond L2 in multilinguals. Among the homogenous set of 16 studies, seven reported whether there were more L1-specific or L2-specific sites in cases where L1 and L2 diverged neuroanatomically in patients with early and/or late age of L2 acquisition (42 cases). Two larger studies investigated bilingual patients (totaling 30 cases) who acquired their L2 early. The studies found more L1- specific regions than L2-specific areas. There were no cases of more L2-specific than L1-specific sites in the early bilinguals.

The results were similar for the complete set of 28 studies. The more divergent sample included ten studies (totaling 89 cases) that reported whether there were more L1-specific or L2-specific regions in cases where L1 and L2 diverged neuroanatomically in patients with early and/or late age of L2 acquisition. The ten studies used ESM, one—fMRI and one—both ESM and fMRI. Four of the ten studies (100%; 73 cases) observed that bilingual patients who acquired their L2 early, had more L1- than L2-specific regions. As was the case with the pool of the homogenous studies, we did not find data in any of the heterogeneous studies that would contradict the result of there being more L1-specific sites, compared to L2-specific regions.

Further, within the set of the 16 homogenous studies, five investigated the prevalence of L1- versus L2-specific sites in patients who acquired their L2 late (12 cases). Among the 12 cases of late bilingualism, 11 displayed more L1-specific areas and two patients had more L2-specific regions.

**Figure 3:**
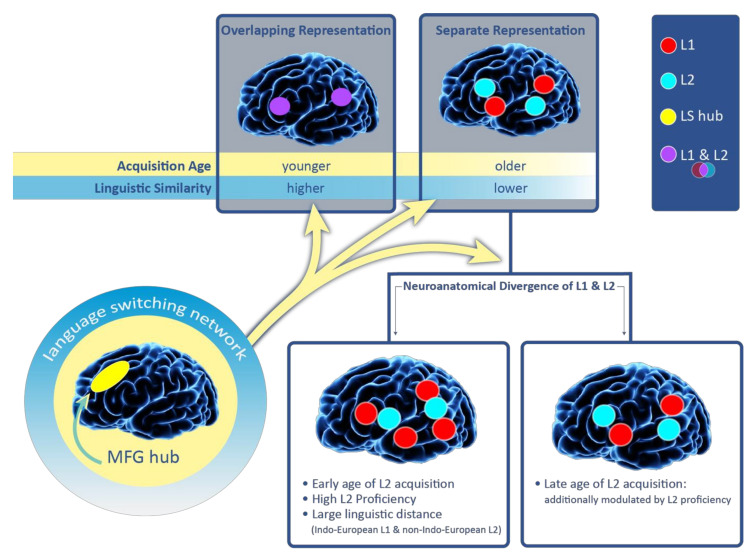
Principles guiding overlapping language representation in bilinguals. A convergent representation of L1 and L2 in the brain is more likely for an early age of L2 acquisition and when the linguistic distance between languages is small (i.e., L1 and L2 are typologically similar). Language switching (LS) is mediated by the language switching network with the middle frontal gyrus (MFG) as the hub. When L1 and L2 diverge neuroanatomically, more L1-specific regions can be expected in: (1) early high proficient bilinguals, (2) individuals with high L2 proficiency and (3) in cases where L1 was an Indo-European language and L2 was non-Indo-European language. The number of L1- and L2-specific regions appears more evenly distributed among late bilinguals and this neural organization might be further modulated by proficiency.

We verified the results for the complete set of 28 studies. The findings were largely similar and are shown in Figure 3 in percentiles. Six studies (totaling 16 cases) examined the prevalence of L1- versus L2-specific sites in patients who acquired their L2 late. We again found 11 cases (69%; five studies) with more L1-specific areas. Five other cases (31%; two studies) showed more L2-specific sites (see Figure 3). In the first of the two studies that observed more L2-specific regions for late bilinguals [29] the authors pointed out that the number of language-specific areas in late bilinguals may be modulated by L2 proficiency. Specifically, one patient in their sample was significantly more L2 proficient compared to the remaining three subjects. This patient had an equal number of L1- and L2-positive ESM, while the other patients had more L2-specific areas. The second of the two studies [39] described a trilingual female patient who was highly proficient in all her languages (only the results for L1 and L3 were reported). She had a glioma within the left prefrontal cortex. There were three L1-positive and four L3-positive sites identified by ESM. Thus the number of language-specific sites was nearly the same. The authors also found a region in the dorsolateral prefrontal cortex (Brodmann Area 9) where ESM stimulation elicited language switching. Importantly, Sierpowska et al. [26,30] found that the middle frontal gyrus mediates the ability to switch between languages. Based on their ESM data and post-operative results, the authors concluded that this region is a hub for a more extended network of areas subserving language switching and cognitive control in bilinguals (see also [6,43,44]). The engagement of the middle frontal gyrus in bilingual patients has been demonstrated intraoperatively [26] but also port-surgically [30]. Specifically, disrupted language switching was reported after tumor resection in the middle frontal gyrus in a bilingual patient. The middle frontal gyrus hub is shown in Figure 3 that summarizes the results of this review.

In sum, the reviewed studies suggest that the age of acquisition of L2 seems to be a robust variable affecting the amount of overlap between L1 and L2. The second language acquired early is more likely to neuroanatomically overlap with L1, while L2 learned late is more likely to be organized separately from L1. Moreover, early L2 acquisition is associated with a greater volume of activation in the right hemisphere for L1 and L2, compared to monolinguals. Finally, we examined the prevalence of L1- versus L2-specific sites in early versus late bilingual and multilingual patients. We analyzed a set of 16 homogenous studies separately and then combined it with a more heterogeneous set of additional 12 studies. Each of the analyses showed that in cases where languages diverged neuroanatomically, more L1-specific areas were found in early bilinguals and multilinguals. Although late bilinguals and multilinguals also displayed considerably more L1 than L2-specific sites, about a third of the reviewed cases had more L2-specific regions. The frequency of occurrence of L1- vs. L2-specific areas may be modulated by proficiency in late bilinguals and multilinguals. These results suggest the importance of mapping both languages in neurosurgical patients who learned their L2 later in life.

#### 3.3.2. Proficiency

The most basic classification of language proficiency has three major levels of proficiency that apply to language production, language comprehension, writing and reading: (a) “low/basic user”—the speaker has basic communicative abilities in L2, (b) “intermediate/independent user”—the speaker can participate in a conversation about familiar matters using connected text and (c) “high/proficient user”—the speaker is very fluent in their L2 and is able to interact with native speakers naturally (based on the Common European Framework of Reference for Languages) [45].

Most of the reviewed papers defined proficiency as low, intermediate or high. Twenty-three reviewed studies explicitly mentioned the level of language proficiency of their participants. In three additional studies, proficiency could be inferred based on patient history with a rather high level of certainty [3,29,32]. In total, of the 256 L2+, proficiency was identified in 227 cases. The vast majority of patients in the reviewed studies had high proficiency in their L2 (*n* = 191), 25 had an intermediate and 11 had low proficiency (see Table 3).

The reviewed sample showed that the level of proficiency did not predict an amount of neuroanatomical overlap between L1 and L2. A high degree of neuroanatomical separation was found both in individuals with high and low L2 proficiency. Note, however, that patients with lower L2 proficiency were underrepresented in this sample.

The remaining paragraphs of this section will collectively report results from bi- and multilingual patients. We will again use the term “L2” to describe L2 in bilinguals and languages beyond L2 in multilinguals. Within the set of 16 heterogeneous studies, there were seven reports (totaling 40 patients) that compared the frequency of occurrence of L1- versus L2-specific sites in individuals with a high L2 proficiency level when L1 and L2 diverged neuroanatomically. Four of the seven studies (38 patients) found more L1-specific regions than L2-specific regions. The two remaining studies (10 patients) demonstrated more L2-specific than L1-specific sites.

**Figure 4:**
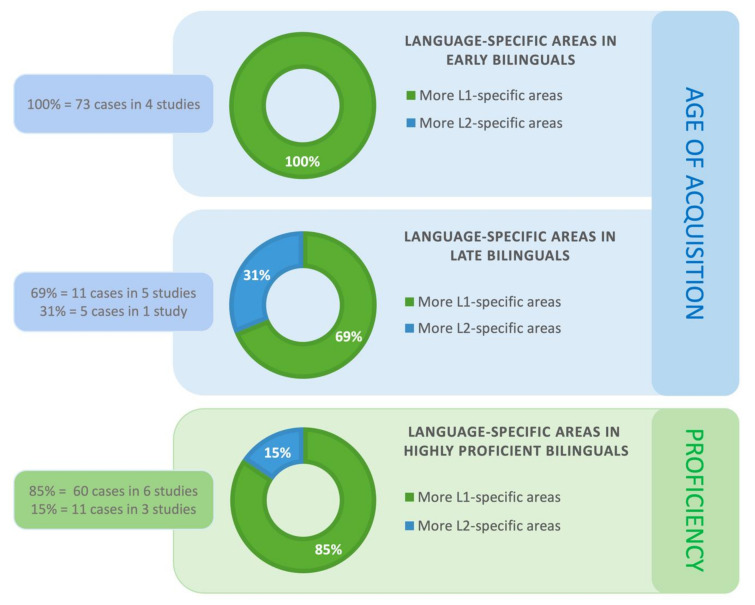
The proportion of studies showing language-specific areas in early and late bilinguals and in highly proficient bilinguals.

The results were confirmed for the complete set of 28 studies. The number of studies that looked at L1- versus L2-specific sites in individuals with a high L2 proficiency level now increased to nine (71 patients). Among these nine studies, six (60 cases; 85%) reported more L1-specific areas and three (11 cases; 15%) found more L2-specific sites. Figure 4 illustrates these findings in percentiles.

The reviewed 28 studies provided only scarce data on the frequency of occurrence of L1- versus L2-specific sites in individuals with a moderate and low L2 proficiency level when L1 and L2 diverged neuroanatomically. Since there were only three case studies with one case per report, we combined moderate and low proficiency of those patients into a single “lower proficiency” category. Because the data on lower proficiency was so limited, we did not present them in Figure 4. One study [32] demonstrated more ESM-positive sites for L1 than L2 in a single patient with lower L2 proficiency. On the other hand, Roux and Tremoulet [25] reported a case with more positive sites for their less fluent L2. However, the authors noted that the craniotomy areas where electrostimulation was applied were restricted [25]. They did not find any differences in the number of L1 versus L2-positive in the remaining five patients examined in that study who had different L2 proficiency levels.

In sum, the degree of proficiency does not seem to predict the amount of neuroanatomical overlap between L1 and L2 in the reviewed studies. Both patients with low and high L2 proficiency had numerous divergent sites subserving L1 and L2. Individuals with high L2 proficiency were more likely to have more L1-specific than L2-specific regions, which was confirmed by both the homogenous and the heterogeneous study set.

#### 3.3.3. The Linguistic Distance between Languages

**Figure 5:**
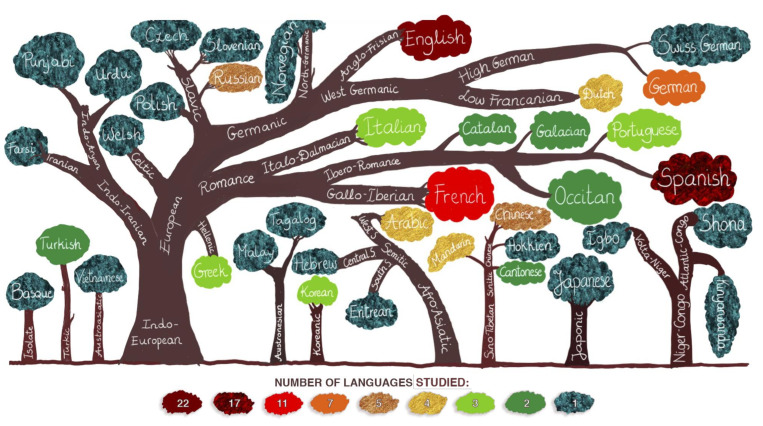
Languages examined in clinical language studies presented with language family trees. The structure of the trees has been simplified in that only languages that appeared in the reviewed studies were included. Different colors correspond to the number of studies in which the languages appeared.

A linguistic distance is a measure of typological similarity between languages. For this review, a linguistic distance was determined using a simple measure: the number of branches between two languages on the language family tree (see Figure 5). In simple terms, a closer distance reflects a high amount of mutual intelligibility and a more considerable distance reflects possible difficulties in communication. For this analysis, we identified four degrees of a linguistic distance: (1) very close—linguistically similar languages that are only one branch apart, for example, German and Swiss German, (2) close—relatively similar languages that are two branches apart, for instance, German and English, (3) moderately close—languages which are three branches apart, such as, for example, German and Spanish and (4) languages that are four (e.g., German and Farsi) or five (e.g., German and Tongan) branches apart [46].

Among the reviewed studies, 40 languages from nine major families were examined (see Figure 5). Additionally, one language (Basque, it appeared in the study by Fernández-Coello et al.) [2] is a language isolate (a language that does not have a genealogical relationship with any other languages) [46]. English appeared in 22 studies, Spanish in 17, French in 11, German in seven, Russian and Chinese in five (“Chinese *” in Figure 5 stands for “unspecified Chinese”; a few of the reviewed studies did not specify which of the Chinese languages were studied), Arabic, Dutch and Mandarin in four and Greek, Korean, Italian and Portuguese in three studies. The remaining languages were assessed in one to two studies (see Figure 5).

We determined the linguistic distance based on the actual languages reported in the reviewed studies. The linguistic distance was assessed between each L1 and each L2+, which totaled 66 language pairs across the reviewed studies. Seven languages were linguistically very close to the patients’ L1, nine were close, 19 were moderately distant and 29 were distant.

There were insufficient data in the homogeneous study set on the effect of the linguistic distance on the amount of neuroanatomical overlap between L1 and L2. We will thus report findings only from the complete set of 28 studies. Rapport et al. [27] performed the Wada test to map languages in multilingual patients. They demonstrated that very close languages (e.g., Mandarin and Cantonese or Mandarin and Hokkien) had a similar recovery during the Wada test using an object naming task. The languages usually recovered before English (a distant L2+). Furthermore, similar (correct or incorrect) responses were more likely between two close languages than a close and a distant language [27]. At the same time, Połczyńska et al. [33] reported no significant difference concerning overlapping versus separate sites between very close (a Swiss German dialect versus German), close (a Swiss German dialect versus English) and moderately close languages (a Swiss German dialect versus French) languages in a quadrilingual patient with a left frontal brain tumor. The authors used fMRI and ESM to map the languages. They found that most overlapping language regions were shared by two languages or more. Only about a quarter of the areas showed co-localization for all the four languages. The authors suggested that the language system in multilinguals shares a small template or arrangement of overlapping language sites within the primary language areas. This case study shows that even though the linguistic distance appears to be an important variable shaping neural representation of languages in multilinguals, linguistically close languages may still be organized separately to some extent [7]. It is also plausible that typologically related languages might require a certain degree of neuroanatomical separation to avoid language interference.

Cheung et al. [21] used fMRI to investigate neural processing during reading in Chinese (L1) and reading in English (L2) in patients with left and right temporal lobe epilepsy, as well as healthy controls. The controls showed left hemisphere-lateralization for English and bi-hemispheric lateralization for Chinese. Both right and left temporal epilepsy patients also showed bilateral hemispheric lateralization for Chinese. Additionally, patients with right temporal epilepsy demonstrated more bilateral organization during reading in English than patients with left temporal epilepsy. It should be pointed out, however, that Rapport et al. [27] who used the Wada test, did not observe additional right hemisphere involvement in multilingual patients who spoke distant languages. It might be that the right hemisphere activity seen with fMRI supports but is not crucial for language function. Therefore it was not observed in the study by Rapport et al. [27] Another explanation could be that the Wada test was not designed to test the likely right hemisphere contributions, including the processing of ideograms during reading. Unfortunately, we are not aware of an ESM report that could weigh in on the additional right hemisphere engagement in non-Indo-European versus Indo-European languages. What has been found, though, are differences between Indo-European and non-Indo-European languages within the left hemisphere using ESM. Namely, the number of L1-specific regions was greater in participants speaking Indo-European languages as the primary language compared to those speaking non-Indo-European languages as the primary language [9].

Taken together, the linguistic distance impacts the cerebral organization of languages. The more distant L1 is from L2+, the more separate neural organization may be expected. More L1-specific regions are predicted when L1 is a language from the Indo-European family and L2 is a non-Indo-European language.

## 4. Discussion

The reviewed data suggest several principles of language organization in bilingual neurosurgical patients. The first major principle is that separate cortical representation uniquely dedicated to each language is the rule rather than an occasional finding. Second, divergent neuroanatomical organization of L1 and L2 is found both in the anterior and posterior language areas. This finding is clinically significant because it highlights the need to map L1 and L2 both anteriorly and posteriorly for surgical purposes. Third, in cases where there was a convergent neuroanatomical representation for L1 and L2, two factors explained the overlap: an early age of L2 acquisition and a small linguistic distance between L1 and L2. On the other hand, when L2 was acquired late and when there is a large linguistic distance between L1 and L2, languages are more likely to be organized separately. This finding suggests the importance of mapping both languages in neurosurgical patients who learned their L2 later and when the linguistic distance between L1 and L2 is larger. Finally, when languages diverge neuroanatomically, there are typically more L1-specific regions in: (1) early, high proficient bilinguals, (2) individuals with high L2 proficiency and (3) in cases where L1 was an Indo-European language and L2 was non-Indo-European language. The number of L1- and L2-specific regions appears more evenly distributed among late bilinguals and this neural organization might be further modulated by proficiency. In sum, this work provides initial evidence-based principles predicting the likelihood of converging versus separate neural representations of L1 and L2 in neurosurgical patients.

Figure 5 illustrates and summarizes the principles presented in this review. We suggest considering these principles when planning neurosurgery in bilingual patients. The principles indicate when it is essential to order pre-surgical fMRI to map all the languages in bilingual neurosurgical patients. Specifically, mapping both languages with fMRI and ESM can be particularly important in patients who learned their L2 later in life and whose L1 and L2 have a larger linguistic distance. Nevertheless, since there were so many instances of separate language organization in all the reviewed studies, we strongly recommend mapping each language in which a patient needs to communicate post-operatively.

Figure 3 also includes the language switching network with the middle frontal gyrus as the hub. The network does not affect language representation in bilinguals and its representation is expected to be similar, regardless of the amount of neuroanatomical overlap. However, the language switching network allows bilinguals to activate one language while presumably silencing or inhibiting the unused language [47]. Thus, the network is our window into assessing the extent of neuroanatomical co-localization and the number of language-specific regions (see the three yellow arrows extending from the language switching network icon in Figure 3).

As can be seen in Figure 3, a younger age of L2 acquisition was associated with more overlapping neural representation. Concurrently, a younger age of L2 acquisition also predicted more L1-specific regions in cases where L1 and L2 diverged neuroanatomically. While the age of L2 acquisition is a robust factor modulating the amount of neuroanatomical co-localization between L1 and L2, we suggest that it is a complex interplay of multiple factors that modulates the extent to which languages converge in the brain. As demonstrated in this study, one such factor can be the linguistic distance between L1 and L2. However, there are likely more factors that contribute to the degree to which two languages overlap in the brain, including the amount of exposure to L1 and L2, whether languages are acquired formally or informally or whether the languages are oral or signed. In fact, prior neuroimaging studies on healthy bilinguals have shown that each of these factors can affect the extent to which languages co-localize [15,48,49]. Each bilingual is unique because of a different interplay of the numerous factors that can modulate language organization. For this reason, generalizations over larger samples of bilinguals have remained challenging [50,51]. Dziubalska-Kołaczyk and Wrembel [51], who discuss the challenges in the context of multilinguals, introduce the term “situation of acquisition”. The term accounts for the unique interplay of multiple factors (e.g., proficiency level, an informal versus formal learning context) in every multilingual speaker in each of the languages they speak (L1 and L2+).

Nearly 16% of the patients examined in this review were multilingual. Differences in the neural organization of languages in bilinguals versus multilinguals are complex and not well understood. Multilinguals have mainly been investigated in case studies [52]. One of the main differences between bi- and multilinguals is that while early bilinguals can be balanced in their languages, early multilinguals will likely be more dominant in some languages than others (i.e., balanced multilingualism is unlikely) [52]. A complicating factor is that languages beyond L2 are more likely to be learned later in life, which also means that they are more likely to be learned formally. The additional differences in how these languages are acquired may contribute to a higher degree of neuroanatomical divergence between L1 (and an early acquired L2 in some cases) and the languages beyond L2. Observations from the current review confirm these predictions. Multilingual patients appeared in eight of the 28 studies. All of the eight studies reported separate cerebral organization for each of the languages spoken by the multilinguals. In addition to the divergent regions subserving separate languages, seven of the eight studies also found shared sites among the multiple languages. The only study that did not find convergent language areas in their multilingual patients (*n* = 7) despite performing a range of language tasks during ESM was a report by Bello et al. [4]. The patients in that study all had brain tumors in the anterior areas of the left hemisphere and were late, highly proficient speakers using their languages daily [4]. It is possible that the late age of language acquisition, coupled with a formal manner of learning, had contributed to the high amount of neuroanatomical divergence between the multiple languages with no regions of co-localization. The strikingly consistent finding of the separate neural organization in the multilingual patients in the reviewed sample highlights the importance of individually mapping each language that the patient needs to communicate in post-surgically.

Because the reviewed studies were heterogeneous in terms of diagnoses, as well as language mapping techniques and tasks used, wherever possible, we first analyzed the data using a homogenous set of 16 studies separately. Next, we verified the findings by adding 12 heterogeneous studies to the set of 16 studies, totaling 28 studies. In all the instances, the pool of the 28 studies confirmed the results from the homogenous study set. However, we note that the different medical conditions included in the heterogeneous study set (brain tumors, AVMs, epilepsy and aneurysms) may impact the results of language mapping to a certain degree. For instance, in patients with pediatric epilepsy and seizure focus in the language-dominant hemisphere (usually left), we frequently observe functional reorganization of language to a contra-lesional hemisphere (usually right) [53]. AVMs [54] and brain tumors [55] may affect language lateralization. Tumor properties, such as type and grade, can also affect language representation in bilinguals. For example, Lubrano et al. [39] observed tissue displacement following a low-grade glioma in a late trilingual patient who spoke German, English and French. The authors noted reshaping attributed to brain plasticity in regions infiltrated by the tumor located in left inferior frontal and prefrontal cortex. The patient had intact language performance 24 months after surgery. Fernández-Coello et al. [2] also found the displacement of language function in three of their subjects with large, slow-growing gliomas in the left language dominant hemisphere. Language function in those patients was not detected near their lesion sites and it was assumed to have reorganized away from the tumor. On the other hand, the authors reported a patient with a small but highly aggressive glioblastoma in whom a functional language area was identified within the cortical border of the extension of the tumor. The authors suggested that in that patient, language function did not have enough time to reorganize away from the lesion. In a similar vein, Połczyńska et al. [33] found that an aggressive brain tumor in the language dominant left frontal gyrus disrupted performance in languages acquired earlier in life (L1 and L2) but not in languages learned in adulthood (L3 and L4). L1 and L2 likely had a more overlapping neural representation than L3 and L4 and because of the aggressive nature of the tumor, the functional areas did not migrate away from the lesion.

Similar to the various diagnoses, different acquisition techniques used in the heterogeneous study set may have influenced the results of clinical language mapping in the reviewed sample. Each of the language mapping methods has its idiosyncrasies [33,56,57]. Functional MRI can guide ESM to make the intraoperative procedure more efficient. For some cases, fMRI may be the only technique available (for certain patients ESM may not be possible) [2,3]. Because fMRI is not invasive and simple, many neurosurgical centers use this method to map language function pre-operatively in patients who need brain surgery. However, the technique may falsely identify certain brain regions as potentially eloquent, while the areas only support but are not critical for language function. Other challenges are smoothing and spatial normalization, as well as the fact that it is hard to have the exact match between fMRI and what the surface of the brain looks like in the operating room. For these reasons, many surgeons also choose to perform ESM. This invasive method often a necessary clinical adjunct to fMRI that is spatially more specific than fMRI. It can clearly identify which areas are essential and which one are simply support a given function but are not eloquent [2,3,57,58,59,60]. For detailed information on the techniques used in each study, see Table 2.

## 5. Limitations

This review has a few limitations that should be considered when interpreting the presented findings on the organization of L1 and L2 in the brain. First, knowing that different language tasks can give different mapping results [20,58], it should be pointed out that the results from the reviewed studies are primarily based on lexico-semantic tasks, while other important language aspects (e.g., syntax) were not examined (see Table 2). Second, while there was insufficient data to review brain activations during specific language tasks, it is known that task differences exist [20,56,61]. The majority of the reviewed studies (*n* = 24) performed an object naming task and 17 of the studies used the task in combination with other language tasks. Those studies rarely reported results for individual tasks. Four studies [22,25,28,35] found task-specific sites. Each of these studies performed an object naming task and a reading task. We therefore strongly support the view that using a panel of tasks in clinical language mapping is superior to using a single task because it is more likely to reflect the complexity of language more accurately than a single task [20,56,61,62,63]. Third, the reviewed studies were heterogeneous in acquisition techniques, patient diagnosis and many aspects of bilingualism unique to each study population; thus, it was not possible for us to conduct a formal statistical meta-analysis. However, when possible, we first analyzed a homogenous data set and then verified the findings for the complete set of 28 studies. We hope that future studies will take account of the various linguistic variables that are important in studying bilingualism, including the age of L2 acquisition, proficiency level of L1 and L2, the amount of language exposure to L1 and L2, the manner of L2 acquisition and the linguistic distance between L1 and L2. We also hope that future studies will report these factors for a more exhaustive understanding of their impact on the neural representation of L1 and L2. Forth, there is a potential for bias in data extraction based on search parameters and keywords used. Fifth, we acknowledge that—while preserving the ability of bilingual patients to communicate in both their languages is important [2,3]—sometimes sacrificing one language may be acceptable to achieve neurosurgical goals. Nevertheless, such intentional decisions need to be considered very carefully and they need to be thoroughly discussed with the patient. Finally, since our results apply to patients with brain lesions who underwent clinical language mapping, we recommend caution in generalizing our results to healthy bilinguals. Following Fernández-Coello et al. [2], we think that while the findings should not be extrapolated to the general population, they are valid for clinicians working with lesions around eloquent language sites in bilingual patients. At the same time, the neuroimaging literature on healthy individuals has reported similar patterns on the effect of the factors on the neural representation of L1 and L2. For instance, there is evidence to suggest that early bilingual controls who acquired their L2 early have both their languages organized more bilaterally than monolinguals [13], which is in line with what has been reported here for bilingual patients [20]. Another example is the literature on the linguistic distance and the amount of neuroanatomical co-localization between L1 and L2 in healthy controls. Numerous studies have demonstrated that typologically more distance languages are more likely to have a shared representation, while typologically closed languages are more likely to have a more similar neuroanatomical distribution [8,64,65].

## 6. Conclusions

The goal of this paper was to review the available single-subject data on bilingual neurosurgical patients to provide an initial set of evidence-based principles predicting the likelihood of converging neural representations of L1 and L2 in neurosurgical patients. We suggest considering these principles when planning neurosurgery in bilingual patients.

## Figures and Tables

**Table 1:** Patient biomedical information.

Study	No. of Patients	Etiology				Lesion Location						Age	Handedness			Gender		Aphasia		Onset Age
		Tumor	Epilepsy	AVM	Aneur	LH	RH	n-L	Ant	Post	N/A		Right	Left	Mixed	Male	Female	Yes	No	
Połczyńska et al., 2017 [20]	25	17	5	3		17	8		14	11		15–83	22	3		14	11	15	10	N/A
Lucas et al., 2004 [9]	22		22			21	1				22	18–53	18	2	2	5	17		22	N/A
Cheung et al., 2009 [21]	21		21			13	8		21			14–48	21			9	12	N/A	N/A	1 to 47
Roux et al., 2004 [22]	19	19				14	5				19	13–76	18	1		N/A *	N/A *		19	N/A
Walker et al., 2004 [23]	17	17				17			9	8		15–57	14		3	11	6	N/A	N/A	N/A
Centeno et al., 2014 *** [24]	16		16			9	4	3	16			24–50	14	2		13	3	N/A	N/A	>1 to 27
Roux & Trémoulet, 2002 [25]	12	12				12			7	5		30–74	12			8	4		12	N/A
Fernández-Coello et al., 2017 [2]	13	13				13			6	6	1	25–62	13			5	8		1	N/A
Gao et al., 2015 ** [7]	11	11				11					11	24–46	11			8	3	1	10	N/A
Sierpowska et al., 2018 [26]	9	9				9			9	1		30–54	N/AA			5	4		9	N/A
Bello et al., 2006 [4]	7	7				7			7			32-58	7			4	3		7	N/A
Rapport et al., 1983 [27]	7	3	1	1	2	5	2		3	3	1	23-45	7			5	2	2	7	21–45, N/A
Borius et al., 2012 [28]	7	7				5	2		5	2		26-45	5	2		3	4		7	N/A
Cervenka et al., 2011 [29]	4		4			4			4			28-50	3		1	3	1		4	8 to 31
Ojemann & Whitaker, 1978 [3]	2		2			1	1		1	1		37, 20	1	1		1	1	N/A	N/A	4, 6
Sierpowska et al., 2013 [30]	2	2				2			1	1		60, 36	2			2		2		36
Kho et al., 2007 [31]	2	1	1			2			1		1	36, 50	2			2			2	7, 44
Bilotta et al., 2011 [32]	1	1				1				1		54	N/A	N/A	N/A		1	N/A	N/A	54
Połczyńska et al., 2016 [33]	1	1				1			1			60	1				1	1		60
Kin et al., 2013 [34]	1	1				1			1			40	1			1			1	40
Pouratian et al., 2000 [19]	1	1				1			1			43	1				1		1	37
Serafini et al., 2008 [35]	1	1				1				1		13	1			1			1	6
O’Grady et al., 2016 [36]	1		1				1		1			33		1			1		1	5
Gomez-Tortosa et al., 1995 [37]	1			1		1				1		22	1				1	1		19
Berthier et al., 1990 [38]	1			1		1				1		25	1			1		1		N/A
Lubrano et al., 2012 [39]	1	1				1			1			31	1				1		1	31
Navarro et al., 2009 [40]	1		1				1		1			34	1			1		N/A	N/A	1
Wang et al., 2013 [41]	1	1				1			1			25	1				1		1	25
TOTAL VALUES	207	1255	74	6	2	171	33	3	111	42	55		179	12	6	102	86	23	116	

AVM = arteriovenous malformation, Aneur = aneurysm, LH = left hemisphere, RH = right hemisphere, n-L = non-lateralizing lesion (in epilepsy cases). * The total number of males and females was only provided for a larger patient sample that also included monolingual participants. ** The authors stated that, out of 11 patients, there were eight males and four females (which totals 12 patients). It was assumed that there were either eight males and three females or seven males and four females. *** Studies including patients with temporal lobe epilepsy. Although the exact lesion location was not specified, temporal lobe epilepsy affects the anterior portion of the temporal lobe in most cases.

**Table 2:** Acquisition techniques and tasks used in clinical brain mapping studies on bilinguals and multilinguals.

Study	Method					Tasks										
	ESM	fMRI	Wada	Other	TOTAL	OBJ	ARN	Read	Count	ActionN	VerbG	Rep	VRN	OBJ S	Other	TOTAL
Połczyńska et al., 2017 [20]		1			1	1	1						1			3
Lucas et al., 2004 [9]	1				1	1										1
Cheung et al., 2009 [21]		1			1			1								1
Roux et al., 2004 [22]	1				1	1		1								2
Walker et al., 2004 [23]	1				1	1										1
Centeno et al., 2014 [24]		1			1						1				1	2
Roux & Trémoulet, 2002 [25]	1				1	1		1								2
Fernández-Coello et al., 2017 [2]	1				1	1										1
Gao et al., 2015 [7]	1				1	1		1	1							3
Sierpowska et al., 2018 [26]	1	1			2	1								1	1	1
Bello et al., 2006 [4]	1				1	1			1	1					1	4
Rapport et al., 1983 [27]	1		1		2	1		1								2
Borius et al., 2012 [28]	1				1	1		1							1	3
Cervenka et al., 2011 [29]	1				1	1										1
Ojemann & Whitaker, 1978 [3]	1				1	1										1
Sierpowska et al., 2013 [30]	1	1			2	1								1	1	3
Kho et al., 2007 [31]	1	1	1		3	1			1		1				1	4
Bilotta et al., 2011 [32]	1				1	1			1	1					1	4
Połczyńska et al., 2016 [33]	1	1			2	1	1						1			3
Kin et al., 2013 [34]	1				1	1	1									2
Pouratian et al., 2000 [19]	1	1		1	3	1										1
Serafini et al., 2008 [35]	1				1	1		1							1	3
O’Grady et al., 2016 [36]		1			1	1		1							4	6
Gomez-Tortosa et al., 1995 [37]	1		1		2	N/A	N/A	N/A	N/A	N/A	N/A	N/A	N/A	N/A	N/A	N/A
Berthier et al., 1990 [38]			1		1	1			1			1			1	4
Lubrano et al., 2012 [39]	1				1	1										1
Navarro et al., 2009 [40]		1			1										1	1
Wang et al., 2013 [41]	1				1	1								1	1	3
TOTAL VALUES	22	10	4	1		24	3	8	5	2	2	1	2	3	15	

ESM = electrocorticography, direct cortical electrical stimulation, OBJ = object naming, ARN = auditory responsive naming, Read = reading, Count = counting, ActionN = verb naming, VerbG = verb generation, Rep = repetition, VRN = visual (reading) responsive naming, OBJ S = object naming with language switching; Other Methods: optical imaging and PET; Other Tasks: used in a single study: Verbal fluency, famous people naming, translation from L2 to L1, letter fluency, L1 reading with L2 responding, synonym generation, sentence comprehension, antonym generation, sentence completion, alphabet recitation, verbal instructions, a semantic judgment of true-false sentences and color/shape naming. N/A = no information was provided on the tasks used.

**Table 3:** Information about factors in the reviewed studies.

Study	L2+ Languages				Proficiency Level				Age of acq.			Manner of acq.			Amount of Exposure				
	L2	L3	L4	L5	High	Int.	Low	N/A	Early	Late	N/A	Form.	Inf.	N/A	Daily	Occ.	Rare	Never	N/A
Połczyńska et al., 2017 [20]	25				25				25				25		25				
Lucas et al., 2004 [9]	22				17		5				22			22					22
Cheung et al., 2009 [21] *	21							21	21					21					21
Roux et al., 2004 [22] **	19	2	1		12	9	1		7	15				22	12		9	1	
Walker et al., 2004 [23]	17	1			18				9	9			3	15					18
Centeno et al., 2014 [24]	16				5	6	5		5	11				16					16
Roux & Trémoulet, 2002 [25]	12	1	1		7	7			4	10				14	7		7		
Fernández-Coello et al., 2017 [2]	13	13	4	1	31				12	19				31	31				
Gao et al., 2015 [7]	11				11				11				11	9	11				
Sierpowska et al., 2018 [26]	9				9				7	2				9					9
Bello et al., 2006 [4]	7	7	5	2	21					21		21			21				
Rapport et al., 1983 [27]	7	6	1	1	9			6		15		10		5	8				7
Borius et al., 2012 [28]	7				7				2	5				7	7				
Cervenka et al., 2011 [29]	4				4					4			1	3	4				
Ojemann & Whitaker, 1978 [3]	2				1	1			1	1			1	1	1				1
Sierpowska et al., 2013 [30]	2				2				2			1	1		2				
Kho et al., 2007 [31]	2				1	1				2		1		1	2				
Bilotta et al., 2011 [32]	1					1				1				1	1				
Połczyńska et al., 2016 [33]	1	1	1		3					3		3			1	1	1		
Kin et al., 2013 [34]	1							1		1				1		1			
Pouratian et al., 2000 [19]	1				1					1		1			1				
Serafini et al., 2008 [35]	1				1				1				1		1				
O’Grady et al., 2016 [36]	1				1					1			1		1				
Gomez-Tortosa et al., 1995 [37]	1				1					1				1	1				
Berthier et al., 1990 [38]	1							1		1		1							1
Lubrano et al., 2012 [39]	1	1			2					2		2			1	1		1	
Navarro et al., 2009 [40]	1				1					1		1					1		
Wang et al., 2013 [41]	1				1					1		1							1
TOTAL values (no. lang.)	207	32	13	4	191	25	11	29	107	127	22	42	44	179	138	3	18	2	96

Int. = intermediate, acq. = acquisition, occ. = occasional, Form. = formal, Inf. = informal, * Early-late age of FL acquisition: 6 years, ** Early-late age of FL acquisition: 7 years. The numbers illustrate instances of languages per each variable (e.g., the number of instances of L2, L3 L4 and L5; the number of instances of high, intermediate and low proficiency levels per each L2+ language). The manner of acquisition and the amount of exposure were based on the information provided in the reviewed studies. Sometimes the information was inferred indirectly. For example, for early simultaneous bilinguals it was assumed that the manner of acquisition was informal. For people who used a given language at work, it was assumed that they used it daily (unless specified otherwise).

## Appendix A. Draft Pubmed Search

1. bilingual AND clinical language mapping
2. bilingualism AND clinical language mapping
3. multilingual AND clinical language mapping
4. multilingualism AND clinical language mapping
5. bilingual AND fMRI AND brain tumor
6. bilingualism AND fMRI AND brain tumor
7. bilingual AND fMRI AND epilepsy
8. bilingualism AND fMRI AND epilepsy
9. bilingual AND fMRI AND arteriovenous malformation
10. bilingualism AND fMRI AND arteriovenous malformation
11. multilingual AND fMRI AND brain tumor
12. multilingualism AND fMRI AND brain tumor
13. multilingual AND fMRI AND epilepsy
14. multilingualism AND fMRI AND epilepsy
15. multilingual AND fMRI AND arteriovenous malformation
16. multilingualism AND fMRI AND arteriovenous malformation
17. bilingual AND Wada
18. bilingualism AND Wada
19. multilingual AND Wada
20. multilingualism AND Wada
21. bilingual AND awake surgery
22. bilingualism AND awake surgery
23. multilingual AND awake surgery
24. multilingualism AND awake surgery
25. bilingual AND direct electrical stimulation
26. bilingualism AND direct electrical stimulation
27. multilingual AND direct electrical stimulation
28. multilingualism AND direct electrical stimulation
29. bilingual AND bilingual AND electrocorticography
30. bilingualism AND electrocorticography
31. multilingual AND electrocorticography
32. multilingualism AND electrocorticography
33. bilingualism AND direct electrical stimulation
34. multilingual AND direct electrical stimulation
35. multilingualism AND direct electrical stimulation
36. bilingual AND EEG AND brain tumor
37. bilingualism AND EEG AND brain tumor
38. bilingual AND EEG AND epilepsy
39. bilingualism AND EEG AND epilepsy
40. bilingual AND EEG AND arteriovenous malformation
41. bilingualism AND EEG AND arteriovenous malformation
42. multilingual AND EEG AND brain tumor
43. multilingualism AND EEG AND brain tumor
44. multilingual AND EEG AND epilepsy
45. multilingualism AND EEG AND epilepsy
46. multilingual AND EEG AND arteriovenous malformation
47. multilingualism AND EEG AND arteriovenous malformation
48. bilingual AND MEG AND tumor
49. bilingualism AND MEG AND tumor
50. bilingual AND MEG AND epilepsy
51. bilingualism AND MEG AND epilepsy
52. bilingual AND MEG AND arteriovenous malformation
53. bilingualism AND MEG AND arteriovenous malformation
54. multilingual AND MEG AND tumor
55. multilingualism AND MEG AND tumor
56. multilingual AND MEG AND epilepsy
57. multilingualism AND MEG AND epilepsy
58. multilingual AND MEG AND arteriovenous malformation
59. multilingualism AND MEG AND arteriovenous malformation
60. bilingual AND TMS AND tumor
61. bilingualism AND TMSAND tumor
62. bilingual AND TMSAND epilepsy
63. bilingualism AND TMS AND epilepsy
64. bilingual AND TMS AND arteriovenous malformation
65. bilingualism AND TMS AND arteriovenous malformation
66. multilingual AND TMS AND tumor
67. multilingualism AND TMS AND tumor
68. multilingual AND TMS AND epilepsy
69. multilingualism AND TMS AND epilepsy
70. multilingual AND TMS AND arteriovenous malformation
71. multilingualism AND TMS AND arteriovenous malformation.
